# Draft Genome Sequences of 12 Clinical and Environmental Methicillin-Resistant Strains of Staphylococcus pseudintermedius Isolated from a Veterinary Teaching Hospital in Washington State

**DOI:** 10.1128/genomeA.00290-18

**Published:** 2018-04-12

**Authors:** Devendra H. Shah, Lisa P. Jones, Narayan Paul, Margaret A. Davis

**Affiliations:** a Department of Veterinary Microbiology and Pathology, Washington State University, Pullman, Washington, USA; b Paul G. Allen School for Global Animal Health, Washington State University, Pullman, Washington, USA

## Abstract

Methicillin-resistant Staphylococcus pseudintermedius (MRSP) is a globally emergent multidrug-resistant pathogen of dogs associated with nosocomial transmission in dogs and with potential zoonotic impacts. Here, we report the draft whole-genome sequences of 12 hospital-associated MRSP strains and their resistance genotypes and phenotypes.

## GENOME ANNOUNCEMENT

Methicillin-resistant Staphylococcus pseudintermedius (MRSP) is an opportunistic canine pathogen that causes infections of the skin and soft tissue, such as superficial or deep pyoderma, wounds, urinary tract, and other body sites, including otitis media or externa, and is often associated with surgical site infections (1, 2). MRSP is also commonly isolated from cats (3–5) and occasionally from rats, cows, and horses (6–8). Hospitalization and antimicrobial treatment are major risk factors for MRSP colonization or infection, making this an important pathogen associated with veterinary hospital-acquired infections (9–16). The number of MRSP/total S. pseudintermedius infections in hospitalized animals at the Washington State University Veterinary Teaching Hospital increased from 1/17 in 2009 to 11/46 in 2017, peaking at 22/71 in 2015; this increase is consistent with recent increases reported worldwide (1). The potential for zoonotic transmission to humans (11, 17–20), multidrug resistance (MDR), and concern that MRSP could be mistaken for methicillin-resistant Staphylococcus aureus (MRSA) (20, 21) suggest the need for improved detection and molecular subtyping tools for epidemiological source tracing of this pathogen, especially during outbreaks. Here, we report draft genome sequences of 12 MDR-MRSP clinical and environmental strains isolated from a veterinary teaching hospital in Washington State (Table 1). A single colony of each MDR-MRSP strain was grown overnight at 37°C in brain heart infusion (BHI) broth (Difco). DNA was extracted using zirconia beads and the Qiagen DNeasy tissue kit (Qiagen, USA), with the exception that additional 70% ethanol washes and RNase incubations were added. Paired-end sequencing libraries (2 × 150 bp) were prepared using NEBNext Ultra II kit (NEB, UK), according to the manufacturer’s protocol, and size selected in the range of 422 to 502 bp (average size, ∼458 bp). All strains were sequenced using the Illumina HiSeq 4000 platform (Illumina, Inc., USA). Sequences were trimmed using BBDuk and *de novo* assembled using Velvet 1.2.10, with the k-mer length set at 99 (22). Contigs were reorganized by aligning to the genome sequence of the reference S. pseudintermedius strain 081661 (GenBank accession no. NZCP016073) (23) using progressiveMauve (24). Automated annotation of the assembled contigs was performed using the NCBI Prokaryotic Genome Annotation Pipeline (https://www.ncbi.nlm.nih.gov/genome/annotation_prok/). Whole-genome multilocus sequence typing was performed using the Staphylococcus pseudintermedius MLST database (https://pubmlst.org/spseudintermedius/), as described previously (25). All of these strains displayed multidrug resistance (resistance to >3 classes of antibiotics) when MICs were measured according to current Clinical and Laboratory Standards Institute (CLSI) protocols (26). The corresponding resistance genes were identified using ResFinder version 3.0 (27). All strains contained staphylococcal cassette chromosome *mec* elements (SCC*mec*), as identified by ResFinder version 3.0. These genome sequences will aid in the development of improved molecular diagnostics and subtyping methods for epidemiological source tracing of MRSP outbreaks. The detailed comparative genomics analysis of these strains is currently ongoing and will be published independently.

**TABLE 1:** GenBank accession numbers for 12 Staphylococcus pseudintermedius strains

Strain	WGS accession no.^*a*^	No. of paired reads	No. of contigs	*N*_50_ (bp)	Genome length (bp)	G+C content (%)	Source	Yr	Resistance profile^*b*^	Resistance genes	MLST^*c*^
200	PHHV00000000	10,091,826	38	134,914	2,620,679	37.4	Skin swab	2011	Ak Aug Am Cfz Vec Cfx Cpd Xnl Cd E Gm Imp Ox P Ti Tim Sxt	*aac(6')-aph(2''), ant(6)-Ia, aph(3')-III, mecA, blaZ, erm*(B), and *dfrG*	64
335	PHHW00000000	10,437,560	51	133,420	2,710,949	37.4	Leg edema	2012	Aug Am Cef Vec Cfx Cpd Xnl Cli Eno E Imp Mar Oxa P Ti Tim Sxt	*aac(6')-aph(2''), aph(3')-III, ant(6)-Ia, mecA, blaZ, erm*(B), and *dfrG*	71
473	PHHX00000000	10,309,238	42	133,290	2,744,531	37.3	Thoracic fluid	2013	Ak Aug Am Cfx Vec Cpd Xnl Cd Eno E Gm Imp Mar Ox Tim Ti Sxt	*aac(6')-aph(2''), aph(3')-III, ant(6)-Ia, blaZ, mecA, erm*(B), and *dfrG*	71
476	PHID00000000	8,007,918	41	133,420	2,745,474	37.3	Thoracic fluid	2013	Ak Aug Am Cfx Vec Cpd Xnl Cd Eno E Gm Imp Mar Ox Tim Ti Sxt	*aac(6')-aph(2''), aph(3')-III, ant(6)-Ia, blaZ, mecA, erm*(B), and *dfrG*	71
586	PHIC00000000	7,874,460	42	151,204	2,665,706	37.4	Urine	2014	Aug Am Cfz Vec Cfx Cpd Xnl Cli Eno E Mar Oxa P Ti Tim Sxt	*aac(6')-aph(2''), aph(3')-III, ant(6)-Ia, blaZ, mecA, erm*(B), and *dfrG*	71
651	PHIB00000000	9,350,720	37	133,419	2,698,461	37.4	Clippers	2015	Ak Aug Am Cfz Vec Cfx Cpd Xnl Cd Eno E Gm Mar Ox P Ti Tim Sxt	*aac(6')-aph(2''), blaZ, mecA, erm*(B), and *dfrG*	71
684	PHIA00000000	7,436,180	49	120,128	2,668,380	37.4	Surgical plate	2015	Ak Aug Am Cfz Vec Cfx Cpd Xnl C Cd Dox Eno E Gm Mar Ox P Ti Tim Sxt	*aph(3')-III, ant(6)-Ia, blaZ, mecA,* and *erm*(B)	84
738	PHHZ00000000	9,172,346	38	171,264	2,640,001	37.4	Clippers	2015	Ak Aug Am Cfz Vec Cfx Cpd Xnl C Cd Eno E Gm Mar Ox P Ti	*aph(3')-III, ant(6)-Ia, aac(6')-aph(2''), blaZ, mecA, erm*(B), *cat*(pC221), and *tet*(M)	45
742	PHHY00000000	10,507,688	37	171,307	2,637,262	37.4	Nasal swab	2015	Ak Aug Am Cfz Cfx Vec Cpd Xnl C Cd Eno E Gm Imp Mar Ox P Tim Ti	*aac(6')-aph(2''), aph(3')-III, ant(6)-Ia, blaZ, mecA, erm*(B), *cat*(pC221), and *tet*(M)	45
424	PRDQ00000000	8,440,642	43	134,451	2,660,157	37.3	Swab	2012	Ak Aug Am Cfz Vec Fox Cpd Cd Eno E Gm Imp Ti Tim Sxt	*aac(6')-aph(2''), aph(3')-III, ant(6)-Ia, mecA, blaZ, erm*(B), *tet*(M), and *dfrG*	924
980	PRDR00000000	7,516,686	45	156,632	2,634,738	37.4	Clippers	2017	Aug Am Cfz Vec Cpd Cf Cd Dox Eno E Gm Min Ox P Te	*aac(6')-aph(2''), aph(3')-III, ant(6)-Ia, blaZ, mecA, erm*(B), and *tet*(M)	45
1019	PRDP00000000	7,956,734	46	133,401	2,737,904	37.2	Abscess fluid	2017	Aug Am Cfz Cpd Cf C Cd Dox Eno E Mar Te Sxt	*aac(6')-aph(2''), aph(3')-III, ant(6)-Ia, blaZ, mecA, erm*(B), *cat*(pC221), *tet*(M), and *dfrG*	930

a WGS, whole-genome sequencing.

b Antibiotic abbreviations: Ak, amikacin; Am, ampicillin; Aug, amoxicillin-clavulanic acid; C, chloramphenicol; Cd, clindamycin; Cf, cefazolin; Cfx, cefoxitin; Cfz, cefazolin; Cpd, cefpodoxime; Dox, doxycycline; E, erythromycin; Eno, enrofloxacin; Gm, gentamicin; Imp, imipenem; Mar, marbofloxacin; Min, minocycline; Ox, oxacillin; P, penicillin; Ti, ticarcillin; Tim, ticarcillin-clavulanic acid; Sxt, trimethoprim-sulfamethoxazole; Vec, cefovecin; Xnl, ceftiofur.

c MLST, multilocus sequence type.

### Accession number(s).

The sequences have been deposited in the NCBI GenBank database. The accession numbers are listed in Table 1.
